# RNA-Seq Reveals Protective Mechanisms of Mongolian Medicine Molor-Dabos-4 on Acute Indomethacin-Induced Gastric Ulcers in Rats

**DOI:** 10.3390/genes13101740

**Published:** 2022-09-27

**Authors:** Terigele Bao, Lan Feng, Sungbo Cho, Hongzhen Yu, Wenjie Jin, Lili Dai, Junqing Zhang, Laxinamujila Bai, Minghai Fu, Yongsheng Chen

**Affiliations:** 1 NMPA Key Laboratory of Quality Control of Traditional Chinese Medicine (Mongolian Medicine), School of Mongolian Medicine, Inner Mongolia Minzu University, Tongliao 028000, China; 2 Hainan Provincial Key Laboratory for Research and Development of Tropical Herbs, School of Pharmacy, Hainan Medical University, Haikou 571199, China

**Keywords:** Molor-dabos-4, gastric ulcer, transcriptomic analysis, mechanism of action, Mongolian medicine

## Abstract

This study aimed to apply transcriptomics to determine how Molor-Dabos-4 (MD-4) protects healthy rats against indomethacin (IND)-induced gastric ulcers and to identify the mechanism behind this protective effect. Rats were pretreated with MD-4 (0.3, 1.5, or 3 g/kg per day) for 21 days before inducing gastric ulcers by oral administration with indomethacin (30 mg/kg). Unulcerated and untreated healthy rats were used as controls. Effects of the treatment were assessed based on the ulcer index, histological and pathological examinations, and indicators of inflammation, which were determined by enzyme-linked immunosorbent assay. Transcriptomic analysis was performed for identifying potential pharmacological mechanisms. Eventually, after identifying potential target genes, the latter were validated by quantitative reverse-transcription polymerase chain reaction (qRT-PCR). After pretreatment with MD-4, gastric ulcers, along with other histopathological features, were reduced. MD-4 significantly (*p* < 0.05) increased the superoxide dismutase (SOD) levels in ulcers and reduced pepsin, TNF-α, and IL-6 levels. RNA-seq analysis identified a number of target genes on which MD-4 could potentially act. Many of these genes were involved in pathways that were linked to anti-inflammatory and antioxidant responses, and other protective mechanisms for the gastric mucosa. qRT-PCR showed that altered expression of the selected genes, such as *Srm*, *Ryr-1*, *Eno3*, *Prkag3*, and *Eef1a2*, was consistent with the transcriptome results. MD-4 exerts protective effects against IND-induced gastric ulcers by reducing inflammatory cytokines and pepsin and increasing the expression of SOD levels. Downregulation of *Srm*, *Ryr-1*, *Eno3*, *Prkag3*, and *Eef1a2* genes involved in regulating arginine and proline metabolism, calcium signaling pathway, HIF-1 signaling pathway, oxytocin signaling pathway, and legionellosis are possibly involved in MD-4-mediated protection against gastric ulcers.

## 1. Introduction

Gastric ulcers are one of the most common upper digestive disorders. An epidemiological survey on peptic ulcers showed that the prevalence of gastric ulcer disease increased from approximately 6 million in 1990 to 8 million in 2019 globally [1]. In China, about 10% of people have suffered from gastric ulcers at some time in their life [2]. An increase in aggressive agents (gastric acid, pepsin, and oxidative stress) and a decrease in protective factors (adhesive mucus, bicarbonate secretion, antioxidant defenses, and blood flow) causes ulcerations [3]. Other etiological variables, including heavy alcohol consumption, sedentary lifestyle stress, and chronic nonsteroidal anti-inflammatory drug (NSAID) use can also contribute to gastric ulcers [4]. In fact, in the latter case, even though NSAIDs are extensively used as analgesics, antipyretics and anti-inflammatory compounds, their use also promotes neutrophil adhesion, damage to the mucosal integrity and gastric ulcerations [5,6,7]. Although proton-pump inhibitors and other synthetic medicines are available for treatment, their adverse effects may cause headache, dizziness, and gastrointestinal symptoms including abdominal pain, constipation, and diarrhea [8,9]. As a result, research has been focused on identifying medicines with greater effectiveness and safety and compounds that confer additional protection against stomach ulcers [10,11].

The use of herbal medications for treating gastric ulcers has been gaining popularity. This is not only because studies involving humans and animal models have shown that their efficacy was equivalent or even better than that of drugs, such as cimetidine and omeprazole [12], but also because ulcer therapies based on herbal medicine tend to be less expensive than chemical-based ones [13,14]. For instance, the South Korean multiherbal formula SR-5 has been reported to considerably decrease the ulcer index in mice, with *agarwood* extracts producing similar effects, along with reduced inflammation, against stomach ulcers in rats [15,16]. Herbal medicines normally act by stimulating the proliferation of mucous cells, triggering antioxidation mechanisms, inhibiting the secretion of gastric acid, and increasing the activity of H(+)/K(+)-ATPases [17], and thus their use can represent a valuable alternative for the effective treatment of gastric ulcers, while limiting the side effects.

Molor-dabos-4 (MD-4) is a Mongolian folk medicinal prescription that is clinically used to treat gastric ulcers, gastroenteritis, and dyspepsia [18]. The recommended clinical dosage for humans is 3 g per day for a patient weighing about 60 kg, and a therapy cycle lasts 21 consecutive days. MD-4 is composed of one mineral (halite) and three medicinal herbs, namely rhizome of *Zingiber officinale* Rosc. (ZOR)*,* seed of *Piper longum* L. (PLL) and fruit of *Terminalia chebula* Retz. (TCR) with equal ratio combinations (Table 1). Halite is composed of NaCl and trace elements including Br, Rb, Cs, Sr [19]. The active substances of ZOR are volatile oil and gingerol; piperlongumine and piperine are proved to be effective components of PLL A number of glycosides and coumarin and phenolic acid have been isolated from TCR, including chebulosides, gallic acid, and chebulic acid. The research data on the chemical composition analysis of MD-4 are limited [20,21,22,23,24]. Based on ethnopharmacological records, MD-4 is also effective for gastric protection, improving digestion and detoxification. However, its mode of action and antiulcer effects are yet to be determined. The current study examined how MD-4 affected indomethacin-induced gastric ulcers in healthy rats by measuring the extent of mucosal damage and the effects of gene expression on inflammation and gastrointestinal functions. Furthermore, using data mining and transcriptomic analyses, the underlying mechanism behind MD-4’s protective effects against indomethacin-induced ulcers were unraveled.

## 2. Materials and Methods

### 2.1. Ethics Statements

Six-week-old male Sprague Dawley rats (SPF grade, batch C-NMG2021012507) of weight 200 ± 20 g were obtained from Liaoning Chang-Sheng Biotechnology Co. (Shenyang, Liaoning, China) The Committee on the Ethics of Animal Experiments at Inner Mongolia Minzu University reviewed and approved the experimental protocols (approval NM-LL-2021-06-15-1), in compliance with the criteria and general principles of the Chinese Experimental Animals Administration Legislation. All surgical procedures were performed after the animals were euthanized using sodium pentobarbital, while minimizing suffering.

### 2.2. Animal Treatment

Thirty-six male rats were allowed to acclimatize for seven days in a controlled environment (12 h light/dark cycle, 22 ± 2 °C, relative humidity of 50% ± 5%) with unrestricted access to water and food. Rats were randomly assigned to six groups: control group, IND group, 0.3, 1.5, and 3 g/kg MD-4 and ranitidine groups (a clinical antiulcer drug). They were then given a 21-day pretreatment as follows: sodium carboxymethylcellulose water solution (CMC-Na, Sigma; 0.5%, St. Louis, MO, USA) was given to the control and IND groups throughout the trial, while MD-4 groups at 0.3, 1.5, or 3 g/kg doses (provided by the Affiliated Hospital of Inner Mongolia Minzu University, Tongliao, China) and ranitidine (30 mg/kg) were suspended in 0.5% CMC-Na solution and provided once daily through gastric administration.

### 2.3. Gastric Ulcer Induction by Indomethacin

The use of IND is a proven method for inducing gastric ulcerations in rats by a single orally administered dose. All rats were subjected to fasting for 24 h prior to the final drug administration. Three hours after providing the last treatment, IND (30 mg/kg) was orally administered to induce gastric ulcerations as previously described [25]. Food and water were then withheld for six hours before eventually injecting pentobarbital intraperitoneally to euthanize the rats. To analyze the effects of treatments, gastric tissues were dissected and Guth’s method [26] was subsequently applied to determine ulcer length and ulcer index. Percentage inhibition was then calculated as follows: inhibition rate = [(UImodel − UItreated)/UImodel] × 100%, where UI is ulcer index.

### 2.4. Pathological and Histopathological Observation

Rats were euthanized as described above to obtain gastric tissues for pathological examinations. In this case, the tissues were fixed for 48 h in 4% paraformaldehyde prior to decalcification in EDTA (10%; Sigma-Aldrich, Darmstadt, Germany), processed and embedded in paraffin. Samples were then sliced into sections of 4 µm thickness and after dewaxing in xylene two times for 15 min and at 37 °C, ethanol of decreasing concentrations was used for rehydrating the tissues. This was followed by a 5 min washing step with distilled water at room temperature before eventually staining the tissues with hematoxylin and eosin (Nanjing Jiancheng Bioengineering Institute, Nanjing, China).

### 2.5. Measurement of Cytokine Levels

Blood collected from the rats was first centrifuged at 4 °C and 4500× *g* for 10 min. The resulting serum samples were then analyzed using ELISA kits to determine IL-6 and TNF-α levels. Washing and subsequent homogenization of tissues in ice-cold 10X Tris buffer (50 mM; pH = 7.4) was followed by centrifugation at 4 °C and 12,000× *g* for 10 min. Prostaglandin E2 (PGE2), superoxide dismutase (SOD), and malondialdehyde (MDA) levels were then determined for the resulting supernatant using commercially available assay kits (Jiangsu Jingmei Bioengineering Institute, Yancheng, China).

### 2.6. Transcriptome Analysis

Six samples of gastric tissues were taken from the rats in the control, IND, and MD-4 treated (3 g/kg) groups and sent for sequencing in BioTree (BioTree, Shanghai, China). The integrity and purity of the extracted RNA were then measured using a NanoPhotometer^®^ UV/vis spectrophotometer (IMPLEN, Westlake Village, CA, USA), and a Bioanalyzer 2100 (Agilent, Santa Clara, CA, United States) respectively. With the help of poly(dT) oligos coupled to magnetic beads, mRNA was purified before being fragmented at high temperature using divalent cations. This was followed by the reverse transcription of fragments using random hexamer primers, with the resulting cDNA strands subsequently adenylated and ligated to NEBNext^®^ adapters with hairpin loop structures. After PCR amplification of the DNA fragments using universal PCR primers, Phusion High-Fidelity DNA polymerase and Index (X) Primer, the products were purified (AMPure XP system), and a library was prepared, with the latter’s quality assessed with an Agilent Bioanalyzer 2100 system. The TruSeq PE Cluster Kit v3-cBot-HS kit (Illumina) was then used for cluster amplification of the samples on a cBot Cluster Generation System prior to sequencing on an Illumina Novaseq platform to generate 150 bp paired-end reads. Eventually, KEGG pathway and GO functional analyses were used to identify genes that were differentially expressed (DEGs) between groups. In this case, an absolute fold change of ≥2 and a corrected *p*-value of 0.05 were selected as thresholds for considering a gene as being differentially expressed.

### 2.7. Validation by Real-Time Quantitative Reverse Transcription PCR (RT-qPCR)

Based on the antiulcer results of MD-4, regulated cytokine levels, and transcriptomic analysis, six key DEGs related to inflammation and oxidative stress from six key KEGG pathways were validated by RT-qPCR to analyze the expression of *Srm*, *Ryr1*, *Eno3*, *Prkag3*, *RPl3l* and *Eef1a2* for the MD-4, IND, and control groups. Using an oligo(dT) primer, a Quantiscript reverse transcriptase (Qiagen, Hilden, Germany) and the same RNA samples that were used for transcriptomics, cDNA strands were synthesized according to the manufacturer’s instructions. The sequences available in GeneBank were also used to design primers for the amplification of the selected genes (Table 2). Eventually, qRT-PCR was carried out on a LightCycler 480 SW 1.5.1 (Roche LightCycler 480 II, Basel, Switzerland) with an initial 10 min denaturation step at 50 °C, followed by 40 amplification cycles, each with denaturation performed for 1 min at 95 °C, prior to a 1 min annealing and extension step at 60 °C. The process concluded with a melt curve obtained through an incremental increase in temperature from 72 °C to 95 °C before finally cooling to 40 °C. The *β-actin* gene was selected as an internal control and gene expression (ΔΔct) relative to the control was determined as a fold change for plotting.

### 2.8. HPLC-MS/MS Analysis of Chemical Constituents of MD-4 Extract

The chemical composition of MD-4 was identified using liquid chromatography- tandem mass spectrometry (LC-MS/MS). MD-4 aqueous extract (145.54 mg) was poured into a 2 mL centrifuge tube, added 1 mL of 70% methanol and 3 mm steel balls, vibrated and crushed with a fully automatic sample rapid grinder (jxfstprp-48, 70 Hz) for 3 min, low-temperature ultrasound (40 KHz) for 10 min, and centrifuged at 4 °C 12,000 rmp for 10 min. The analysis was performed on a Thermo Ultimate 3000 LC-MS System (Waltham, MA, USA). The column is Zorbax eclipse C18 chromatographic column (1.8 μm × 2.1 × 100 mm) operated at 40 °C. The elution solvents were aqueous 0.1% formic acid (A) - acetonitrile (B). Samples were eluted using a linear gradient from 0–2.0 min, 5% B; 2.0–6.0 min, 30% B; 6.0–7.0 min, 30% B; 7–12 min, 78% B; 14–17 min, 95% B; 17–20 min, 95% B; 20–21 min, 5% B; and 21–25 min, 100% B. The flow rate was 0.3 mL/min. Mass spectrometry was performed using full scan (*m*/*z* 100~1500) and data-dependent secondary mass spectrometry scanning mode (dd-ms2, topN = 10). Primary mass spectrometry resolution was set at 120,000, secondary mass spectrometry resolution was 60,000. Collision mode was set as high-energy collision dissociation and ion heater temperature was 325 °C. Gas flow rate was 45 arb and auxiliary air velocity was 15 arb. Electric spray voltage was 3.5 kV and S-lens RF level was 55%. The chemical structures of MD-4 were characterized based on their retention behavior and MS information, and from reference to databases such as Scifinder and Chemspider, as well as the general literature.

### 2.9. Statistical Analysis

All results were provided as means ± standard error of mean (SEM). Using the GraphPad Prism 5^®^ software (GraphPad software, San Diego, CA, USA), one-way analysis of variance (ANOVA) and LSD tests were then performed to analyze differences between means at 5% significance levels.

## 3. Results

### 3.1. Effects of MD-4 on IND-Induced Gastric Ulcers

**Figure 1:**
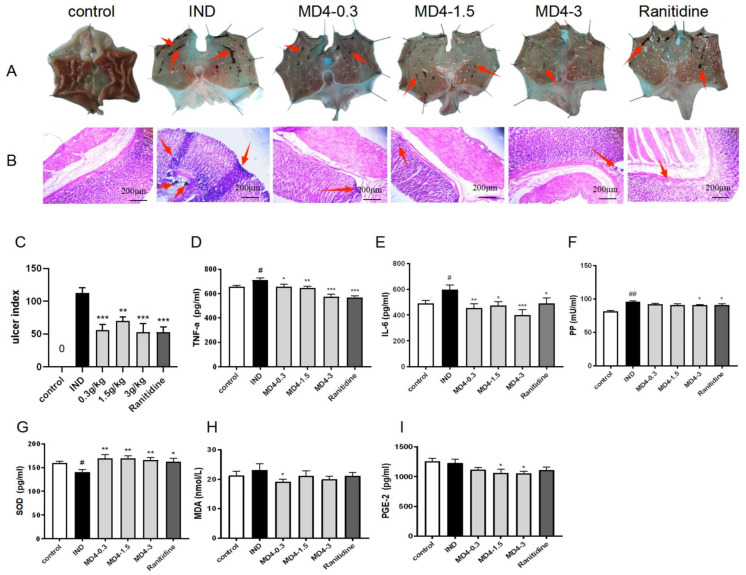
(**A**,**B**): Histopathological features after MD-4 treatment on IND-induced gastric ulcers in rats. In the IND group, rats showed severe injury and inflammation of the gastric epithelium and edema of submucosa. MD-4 improved these alterations dose-dependently and showed less mucosal damage and milder inflammation in contrast to the IND group. IND: indomethacin; MD-4: molor dabos-4 decoction. (**C**): Ulcer index. Data are reported as means ± SEM (*n* = 6). ** *p* < 0.01 vs. IND group; *** *p* < 0.001 vs. IND group. (**D**–**I**): Levels of TNF-α, IL-6 and pepsin in serum and SOD, MDA and PGE-2 in gastric tissues. Data are expressed as means ± SEM (*n* = 6). ^#^ *p* < 0.05 vs. control group; ^##^ *p* < 0.01 vs. control group; * *p* < 0.05 vs. IND group; ** *p* < 0.01 vs. IND group; *** *p* < 0.001 vs. IND group.

Macroscopic observations showed that, unlike the control for which hemorrhages were practically absent from gastric tissues, the IND-treated group displayed ulcerations and hemorrhagic lesions on the stomach’s mucosal layer. In contrast, both MD-4 and ranitidine reduced ulcerations in the treated groups, with the greatest change in terms of increased inhibition rate and decreased ulcer index being 53.27% after treatment with MD-4 at a dose of 3 g/kg. Histopathological observations indicated that, in addition to serious damage to epithelial tissues, the IND group also had necrotic lesions and extensive edematous submucosal layers, all of which were clear indications of ulcerations. MD-4- and ranitidine-treated groups showed less mucosal damage and milder inflammation in contrast to the IND group (Figure 1A–C).

### 3.2. Modulation of Inflammatory and Oxidative Processes by MD-4 in IND-Induced Ulcers

IND administration significantly increased the levels of IL-6, TNF-α and PP compared with the controls (*p* < 0.05), as shown in Figure 1D–F, while treatment with MD-4 significantly decreased their concentrations (*p* < 0.05). In contrast, Figure 1G shows that the IND- and MD-4-treated groups, respectively, showed a decrease and an increase in the SOD levels of gastric tissues (*p* < 0.05). Although IND did not influence MDA or PGE-2 levels in gastric tissue, MD-4 reduced MDA (at 0.3 mmol/L) and PGE-2 (at 1.5 and 3.0 mmol/L) at certain doses (Figure 1H,I).

### 3.3. Altered Gene Expression by MD-4 in Ulcerated Rats

**Figure 2:**
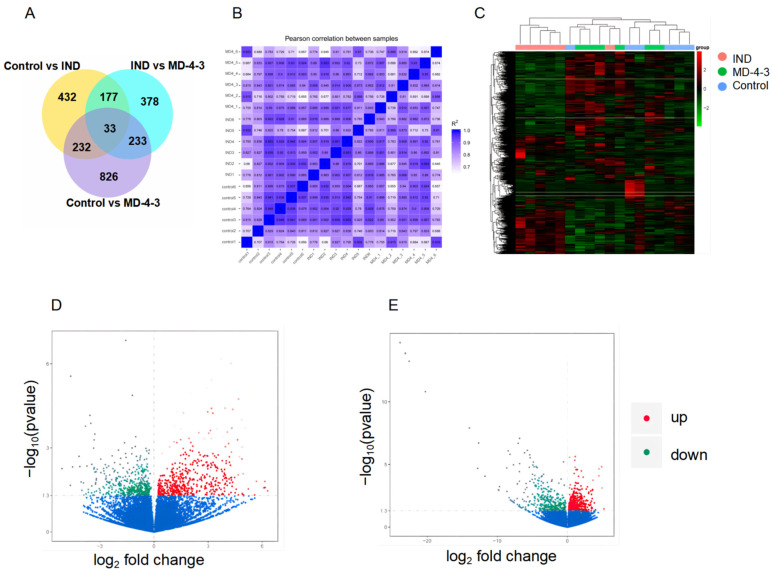
Transcriptomic analysis of MD-4-treated IND-induced gastric ulcerated rats. (**A**): Venn diagram shows the number of differentially expressed genes. Different colors represent different comparisons, adding all the numbers in one circle provides the number of differentially expressed genes for a particular comparisons, while overlapping areas represent common differentially expressed genes when two groups were compared. (**B**): Pearson correlation heat map between samples. The abscissa and ordinate are the square of the correlation coefficient of each sample. (**C**): Cluster heat map of genes that were differentially expressed. The abscissa indicates sample names, with the ordinate being the normalized FPKM vale. Red and green colors indicate upregulation and downregulation, respectively. (**D**,**E**): Volcano plots showing transcriptome data. (**A**,**B**): 874 and 821 genes were differentially expressed when comparing the control with IND treatment (**A**) and between IND- and MD-4-treated groups (**B**), respectively. Vertical lines indicate a log2 fold change, while the horizontal ones represent *p*-value of 0.05. Red and green colors indicate significant up- and downregulation of genes, respectively. Significant DEGs were identified based on a *p*-value of <0.05 and a log2 fold change of at least 2.0. Control: normal control, IND: indomethacin, MD-4: molor-dabos-4 decoction.

The results of sequencing data quality showed that the base quality of sequences was above Q20 and there was no base shift (Figure 2A). The sample correlation heat map showed the gene expression of the three groups showed intragroup correlation (Figure 2B). A Venn diagram showed that 210 out of 821 DEGs responding to GU treatment were related to DEGs caused by MD-4 (Figure 2C). Hierarchical clustering analysis showed that in each group DEGs expression was dramatically different (Figure 2D). An analysis of the number of DEGs in ulcerated gastric tissues and subsequent volcano plots showed that 874 genes (403 downregulated and 471 upregulated) were differentially expressed between the control and the IND-treated groups while a comparison of the IND and MD-4 groups revealed 821 DEGs (388 downregulated and 433 upregulated) (Figure 2E,F).

### 3.4. KEGG Pathway Analysis

**Figure 3:**
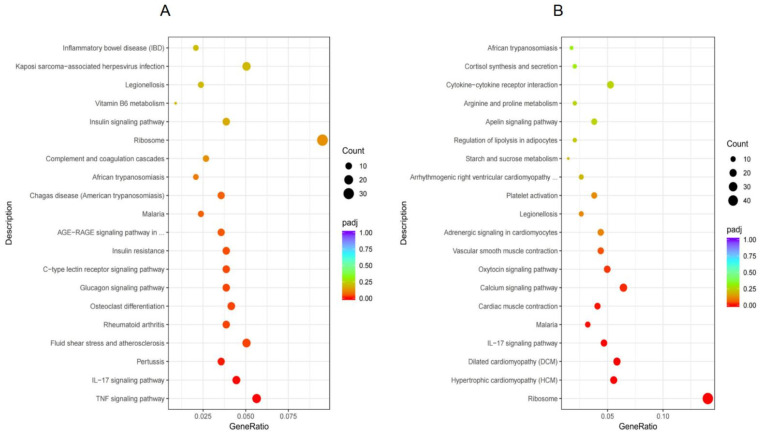
KEGG pathway enrichment analysis. The abscissa is the ratio of the number of differential genes annotated to the KEGG pathway to the total number of differential genes, and the ordinate is the KEGG pathway. (**A**): control vs. IND, (**B**): IND vs. MD-4.

RNA-seq data were analyzed with R software, along with the KEGG database, to identify enriched pathways after treatment with IND and MD-4. Results of KEGG enrichment analysis indicated that genes that were differentially expressed between the control and the IND-treated groups (Figure 3A) were mostly enriched for pertussis, fluid shear stress, atherosclerosis, rheumatoid arthritis, osteoclast differentiation, TNF, IL-17, glucagon, type C lectin receptor, and AGE-RAGE signaling pathways (padj < 0.05). On the other hand, when comparing the IND- and MD-4-treated groups (Figure 3B), the DEGs were mainly enriched for ribosome, hypertrophic cardiomyopathy (HCM), dilated cardiomyopathy (DCM), malaria, cardiac muscle contraction, vascular smooth-muscle contraction, and the IL-17, calcium, and oxytocin signaling pathways (padj < 0.05). Overall, six pathways involving 18 DEGs were common to the IND- and MD-4-treated groups when compared to the control and these included arginine and proline metabolism, ribosome, legionellosis, and the HIF-1, oxytocin, and calcium signaling pathways. Interestingly, patterns of gene expression followed a similar but opposite trend for the IND- and MD-4-treated groups, as the upregulation of one gene observed for one group was matched with the downregulation of the same gene in the second one (Table 3).

### 3.5. qRT-PCR Validation

**Figure 4:**
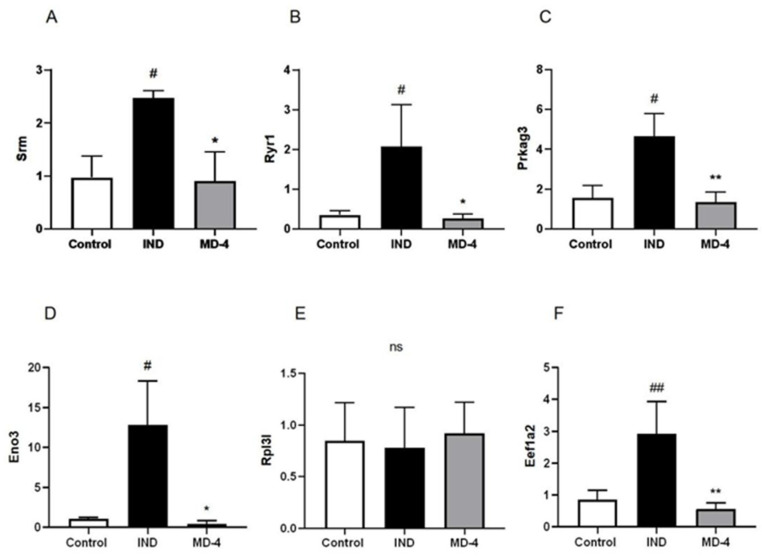
Relative expression of *Srm*, *Ryr1*, *Prkag3*, *Eno3*, *Rpl3l* and *Eef1a2* determined by qRT-PCR. *β-actin* was selected as an internal control. Values represent means ± standard error mean (*n* = 6). ^#^ *p* < 0.05 vs. control, ^##:^ *p* < 0.01 vs. control. * *p* < 0.05 vs. IND-treated, ** *p* < 0.01 vs. IND-treated.

The MD-4 mediated changes in the expression of *Srm*, *Ryr1*, *Eno3*, *Eef1a2*, *Rp131* and *Prkag3* genes that could actually be related to anti-inflammatory or antioxidant responses were selected for further validation by qRT-PCR. Changes in the expression patterns of three out of five selected genes were consistent with the transcriptome results (Figure 4). Expression of *Srm*, *Ryr1*, *Eno3*, *Eef1a2* and *Prkag3* mRNA were increased in the IND group and decreased by MD-4 treatment. No similar expression patterns were observed in the *Rp131* gene.

### 3.6. Chemical Components of MD-4 Extract

All compounds detected by HPLC-MS/MS and provided accurate relative molecular weight compared with the references. A total of 12 compounds were identified with an inclusion rate above or closer to 1%. Piperine and gallic acid were the two main compounds, accounting for 23.57% and 12.02%, respectively. The total inclusion rate of the 12 identified compounds reached 61.14% in MD-4 extract (Table 4).

## 4. Discussion

Previous studies have reported the therapeutic benefits of MD-4 against functional dyspepsia and fatty liver [27] and similar effects against ulcers, and the underlying mechanism of action are yet to be thoroughly investigated. Thus, this study aimed to investigate whether MD-4 could display protective effects against IND-induced gastric ulcers in a rat model while uncovering the mode of action based on transcriptomic approaches. The results of histopathological examinations showed that MD-4 provided significant protection against gastric mucosal hemorrhage and inflammation. Mucosal proliferation is important for healing ulcers. According to research, MD-4 composition also has an antiulcer effect. For example, ginger has a protective effect on acute gastric ulcers in rats. The protective mechanism may be to enhance the gastric defense mechanism, gastric antioxidant capacity, and anti-inflammatory capacity by increasing mucosal PGE2 [28]. Pepsin also causes an imbalance between the protective and invasive factors of the mucosal layer, resulting in the corrosion of gastric mucosa [29]. The current data showed that IND induced acute gastric ulcers in rats by upregulating pepsin, which is consistent with previous reports [30], while MD-4 decreased pepsin level in the gastric tissue, thus suggesting that MD-4 protected the gastric mucosa by inhibiting pepsin levels and reducing risks of gastric ulcer recurrence.

MD-4 was demonstrated to protect against oxidation in cases where cells increased the production of antioxidant factors such as SOD, HO-1, and GSH and reduced MDA level [31,32]. However, the ability of MD-4 to regulate inflammatory factors as an antioxidant mechanism has not yet been reported. TNF-α contributes to gastropathy induced by *Helicobacter pylori*, alcohol consumption and NSAIDs, with studies carried out on NSAID-treated rats showing that it can increase the expression of adhesion molecules, regulate apoptosis in gastric epithelial cells, and promote neutrophil adherence [6]. The results of this study showed that, compared with the IND-treated group, MD-4 significantly reduced TNF-α and IL-6 levels, thus indicating that it could exert antiulcer effects through anti-inflammatory activity. The continuous production of reactive oxygen species (ROS) under normal conditions is balanced by their rapid removal through the body’s antioxidant mechanisms. However, an imbalance in this process can result in gastric damage [33], and therefore synthetic or herbal sources of drugs that scavenge free radicals can significantly protect tissues against oxidative damage and enhance wound healing by decreasing, deactivating or eliminating ROS [34]. Given that gastric ulcers represent one of the damages caused by oxidative stress, it is believed that antioxidant mechanisms may also mediate inhibitory effects on indomethacin-induced gastric ulcers [35]. In this context, studies on humans and animal models have suggested that a number of mechanisms could be involved in the observed effects of herbal medicines on ulcers and these include antioxidant activities, increased production of mucus, stimulated proliferation of mucosal epithelial cells, reduced inflammation and reduced production and secretion of acid [36]. While decreased levels of SOD have been associated with indomethacin-induced gastric ulcers [37], this study showed that MD-4 could increase its levels in gastric tissues, thereby suggesting that MD-4 can protect gastric mucosa by regulating SOD results, as shown in this study. Histological examination further indicated that, in addition to reduced ulcer sizes and ulcer formation, treatments with MD-4 also minimized congestions, hemorrhages and mucosal necrosis. Altogether, the above results highlight the potential mechanism behind the antiulcer effects of MD-4 in IND-treated rats.

Halite, as the main component of MD-4, is a natural mineral salt that aids digestive functions. Similarly, it has been reported that within the gastrointestinal environment, bismuth salts, including salicylate, can protect the gastric mucosa, form a demulcent film that provides quick relief against irritation or even adhere to mucus and ulcerative lesions, along with bile acids, to provide a protective coating [38,39]. Other natural compounds include ZOR (ginger) extracts, whose active constituents have been shown to possess antioxidant, anti-inflammatory and antitumor properties in in vitro and in vivo studies [40,41]. In addition, a previous study reported the antibacterial and antioxidant effects of TCR [42] while Hu et al. discovered that PLL could not only exert anti-inflammatory effects against human osteoarthritis chondrocytes but also reverse the inhibitory effects of IL-1β on cell viability [43]. Although only 0.72% inclusion of 6-shagaol was found in MD-4, it has been reported that 6-shagaol is the main active compound of ginger and exerted significant anti-inflammatory and antioxidant actions that promoted mucosal repair of ulcerated rats [44]. In addition, the contents of piperine and piperidine were identified as 23.54% and 4.43% respectively, which were proved to be anti-inflammatory and antioxidant components in PLL and ginger [45,46]. Gupta et al. showed that gallic acid had antiulcer effects through antioxidation, and the results of this study showed that the content of gallic acid in MD-4 was 12.02% [47]. The findings of this study thus support the above studies by suggesting that the compounds present in MD-4 could display protective effects mainly by modulating inflammatory and antioxidant mechanisms.

Transcriptomics is useful to better understand the biological effects of traditional compounds and reveal their targets or underlying mechanisms of action [48]. In this context, NF-κB and IL-17 are responsible for regulating genes that are linked to inflammatory responses [49], with studies revealing that biochemical factors, including IL-17 levels, were significantly increased in ulcerated rats [50]. Similarly, the IL-17 signaling pathway regulates both the development and the recovery of stress gastric ulcers in a coordinated manner [51]. In addition, a number of inflammatory cells express the receptor for AGE and when the latter gets activated by AGE, this causes many downstream signaling pathways to be activated, with these processes leading to impaired inflammatory responses [52]. VEGF, a specific mitogen of endothelial cells, also regulates angiogenesis [53] and this process was also found to contribute to the healing of ulcers in rats after VEGF treatment. In an experimental model of colitis and gastric injury, oxytocin was found to have a protective effect on the gastrointestinal system. Studies have shown that this compound reduces inflammatory processes by limiting oxidative stress associated with mitochondrial dysfunctions and regulating IL-6 and TNF-α levels [54].

Analysis of gene expression profiles after treatment with MD-4 resulted in the identification of 874 genes for IND-induced ulcers, while 821 genes were found in the case of rats treated with 3 g/kg of MD-4. Many of these genes could actually be related to anti-inflammatory or antioxidant responses. For instance, *Srm* (spermidine synthase) was downregulated after MD-4 treatment. Spermidine, as a polyamine that is present in living tissues and ribosomes, has a number of metabolic functions, with evidence pointing to its role in other mechanisms such as lipid metabolism, reduced inflammation and controlled cell growth, proliferation, and apoptosis [55]. In fact, an increase in spermidine levels tends to be associated with decreased NO levels [56]. Similarly, ryanodine receptor 1 (*Ryr-1*), another protein that is mostly present in skeletal muscles and referred to as the skeletal muscle calcium release channel or the skeletal muscle-type ryanodine receptor [57], is also linked to vasodilation, antioxidation, and gastric cancer [58,59]. In addition to *Ryr-1*, this study found a similar trend for *TrDn*, which is known to interact with *Ryr-1* to regulate the latter’s expression, along with the activity of Casq [60]. The identified DEGs also included the *Eno3* gene, which encodes one type of mammalian enolase isoenzyme known as enolase 3 or β-enolase. In this case, even though *Eno3′s* expression is affected by oxidative stress [61], the gene can also induce inflammatory responses [62]. Another potential target of MD-4 is the *Prkag3* gene, which is linked to oxidative stress and SOD activity through the AMP-activated protein kinase (AMPK) signaling pathway by encoding a regulatory subunit of the AMPK enzyme [63,64]. The function of the elongation factor-1 complex is to deliver aminoacyl tRNAs to the ribosome, and given that one isoform of its α subunit is encoded by the differentially expressed *Eef1a2* gene, this would justify why “ribosome” was identified as one of the enriched pathways. Studies have shown that *Eef1a2* was related to increased IL-6 levels and the occurrence of gastric cancer [65], while Zhou et al. not only demonstrated the increased expression of *Eef1a2* in gastric cancer but also that this gene could act as an independent risk factor to predict prognosis for this condition [66].

In addition, the significant differences in KEGG pathways also showed that inflammatory responses and those involved against oxidative stress may protect against IND-induced gastric ulcer. These pathways included arginine and proline metabolism, oxytocin signaling, HIF-1 (hypoxia inducible factor 1) signaling and calcium signaling. and it is likely that they could be the major ones involved in the chrysomycin A treatment of neuroinflammation injury. Arginine and proline metabolism are related to enzymes that use coenzyme Q10 as electron acceptors [67], and the latter is often viewed as an important endogenous antioxidant [68]. Furthermore, Wang et al. demonstrated that triterpenoids had antiulcer effects on gastric ulcers in rats due to their endogenous metabolites that were related to the calcium pathway [69]. Finally, under hypoxic conditions, HIF-1 levels increase to promote angiogenesis by regulating the expression of related genes [70]. Thus, the HIF-1 signaling pathway plays an important role in metabolic adaptation to hypoxic stress [71]. Hypoxia generates reactive oxygen species, leading to oxidative stress, which is known to activate the transcription of genes involved in promoting angiogenesis. HIF-1 can be stably expressed only under hypoxic conditions. In the current study, *Eno3* (enriched HIF pathway) was significantly upregulated in IND rats, and this expression trend was also validated by qRT-PCR. Thus, oxidative stress and angiogenesis may be one of the important causes of IND-induced gastric ulcers. The significant downregulation of *Eno3* in the MD-4 group indicated that pretreatment could prevent gastric ulcers caused by oxidative stress in rats. MD-4 is currently used clinically to treat symptoms such as indigestion. This study proved the role of MD-4 in preventing gastric ulcers in experimental animals, and revealed that its mechanism is to reduce oxidative stress by downregulating key genes, such as *Eno3*. Therefore, in the clinical use of NSAID, it is recommended to take MD-4 in advance to reduce the symptoms of gastric ulcer. In addition, the next study should design clinical tests to further investigate the antiulcer effect of MD-4.

Our data demonstrate that MD-4 protected against indomethacin-induced gastric ulceration by reducing pepsin activity and inflammatory cytokines and increasing gastric SOD secretion. We propose that regulation of *Srm*, *Ryr-1*, *Eno3*, *Prkag3* and *Eef1a2* genes involved in regulating arginine and proline metabolism, calcium signaling pathway, HIF-1 signaling pathway, oxytocin signaling pathway and legionellosis are may be involved in mechanisms by which MD-4 protects against gastric ulcers. The Mongolian medicine prescription MD-4 is a promising gastroprotective agent with potential use for treating gastric ulcers in clinical practice.

## Figures and Tables

**Table 1:** Components of MD-4.

No	Genus Species	Common Names	Plant Part	Content (%)
1	Halite	Salt	-	25
2	ZOR	Ginger	Rhizome	25
3	PLL	Piper longum	Immature fruits	25
4	TCR	Chebulae Fructus	Fruits	25
Total content (%)				100

**Table 2:** Primers used for qRT-PCR to determine gene expression in gastric tissues.

Gene	Accession No.	Forward Primer	Reverse Primer
*Srm*	NM_053464	ACTCTTGCCCACCAACCAAG	TTGTTGGGTCACAGGGCATAG
*Ryr1*	XM_001078539	CTGAGCTGAATGAATACAACGC	CCATGAGCCTTTCTAGCACTG
*Eno3*	NM_012949	CTGATGACTCTTCCAGCCTC	ACACTTAGTTTCTTTTCCAGCA
*Prkag3*	NM_001106921	AGTCTGCAGGAAACATCGCT	CTCTCTCTGCATTGGACCCC
*Rpl3l*	NM_005061.3	GCTGGCACCAAGAAGAGAGT	AGCATCCGTGGCCAAACTTA
*Eef1a2*	NM_012660	CGGTATCCTCCGTCCTGGTA	CGGCGAATGTCCTTGACAGA
*β-actin*	NM_031144.3	GGAGATTACTGCCCTGGCTCCTA	GACTCATCGTACTCCTGCTTGCTG

**Table 3:** Main genes involved in the six major enriched Kyoto Encyclopedia of Genes and Genomes pathways common to IND- and MD-4-treated groups.

KEGG Pathway	Gene Symbol	Official Full Name	Log2 Fold Change		GeneBank Accession No.
			Control vs. IND	IND vs. MD-4	
Arginine and proline metabolism	*Ckm*	creatine kinase, M-type	+2.894	−3.376	NM_012530
	*Srm*	spermidine synthase	+2.146	−2.099	NM_053464
Calcium signaling pathway	*Casq1*	calsequestrin 1	+3.041	−2.955	NM_001159594
	*Ryr1*	ryanodine receptor 1	+3.152	−4.929	XM_039100854
	*AABR07005775.1*	Rattus norvegicus strain mixed contig_5872, whole genome shotgun sequence	+4.672	−8.455	AABR07005775
	*Hrc*	histidine rich calcium binding protein	+2.700	−2.963	NM_181369
	*Mylk3*	myosin light chain kinase 3	+3.985	−4.653	NM_001110810
	*Tnnc2*	troponin C2, fast skeletal type	+2.653	−4.315	NM_0010373510
	*Trdn*	triadin	+2.934	−3.886	NM_021666
	*Mylk2*	myosin light chain kinase 2	+4.898	−7.565	NM_057209
HIF-1 signaling pathway	*Eno3*	enolase 3	+2.209	−2.069	NM_012949
Oxytocin signaling pathway	*Cacng6*	calcium voltage-gated channel auxiliary subunit γ 6	+3.491	−2.382	NM_080694
	*Prkag3*	protein kinase AMP-activated non-catalytic subunit γ 3	+4.891	−4.264	NM_001106921
	*Ryr1*	ryanodine receptor 1	+3.152	−4.929	XM_039100854
	*Mylk3*	myosin light chain kinase 3	+3.985	−4.653	NM_001110810
	*Mylk2*	myosin light chain kinase 2	+4.898	−7.565	NM_057209
Ribosome	*Rpl3l*	ribosomal protein L3 like	+3.479	−4.562	NM_001191589
Legionellosis	*Eef1a2*	eukaryotic translation elongation factor 1 α 2	+2.396	−2.477	NM_012660

**Table 4:** HPLC-MS/MS identification of MD-4 aqueous extract.

	Chemical Name	Formula	Theoretical Value	Test Value	Content
1	Piperine	C_17_H_19_NO_3_	285.13	285.13	23.54%
2	Gallic acid	C_7_H_6_O_5_	170.02	170.02	12.02%
3	3-(3,5-Dinitrophenyl)-2-methyl-4(3H)-quinazolinone	C_15_H_10_N_4_O_5_	326.06	326.06	5.10%
4	Piperinine	C_17_H_21_NO_3_	287.15	287.15	4.43%
5	MLS002473214-01!(2E,4E)-5-(1,3-benzodioxol-5-yl)-N-(2-methylpropyl)penta-2,4-dienamide	C_16_H_19_NO_3_	273.13	273.13	3.49%
6	4,5-Dinitro-9-oxo-9H-fluorene-2,7-dicarboxamide	C_15_H_8_N_4_O_7_	356.03	356.03	3.16%
7	1,3,6-Trigalloyl glucose	C_27_H_24_O_18_	636.09	636.09	2.63%
8	Methanediol,di-p-toluenesulfonate	C_15_H_16_O_6_S_2_	356.03	356.03	1.93%
9	D-(+)-Galactose	C_6_ H_12_O_6_	180.06	180.06	1.47%
10	4-{[(7-Isopropyl-1,4a-dimethyl-1,2,3,4,4a,9,10,10a-octahydrophenanthren-1-yl)methyl]amino}-4-oxobut-2-enoic acid	C_24_H_33_NO_3_	383.24	383.24	1.42%
11	1,6-Bis-O-(3,4,5-trihydroxybenzoyl)hexopyranose	C_20_H_20_O_14_	484.08	484.08	1.23%
12	6-shogaol	C_17_H_24_O_3_	276.17	276.17	0.72%

## Data Availability

Data are contained within this article.
